# Diagnosis of Peripheral Facial Palsy Associated with Parvovirus B19 Infection by Polymerase Chain Reaction

**DOI:** 10.1155/2022/4574640

**Published:** 2022-01-10

**Authors:** Taro Fukuta, Yoshihiko Kawano, Maiko Ikeda, Jun-ichi Kawada, Yoshinori Ito, Shinya Hara

**Affiliations:** ^1^Department of Pediatrics, Toyota Memorial Hospital, 1-1 Heiwa-Cho, Toyota, Aichi 471-8513, Japan; ^2^Department of Pediatrics, Nagoya University Graduate School of Medicine, 65 Tsurumai-Cho, Nagoya, Aichi 466-8560, Japan

## Abstract

Human parvovirus B19 (PVB19) infection causes neurological manifestations, including encephalitis, meningitis, and neuropathy, but facial nerve palsy is rare. Moreover, no case of facial nerve palsy related to PVB19 infection that was diagnosed by PCR and serology has been reported. A 19-month-old boy without the medical history developed facial nerve palsy and was treated with prednisolone and valacyclovir. On the 19th day, erythema appeared on his body, and the PVB19-specific IgM and PVB19 DNA were detected in the serum, leading to the diagnosis of infectious erythema associated with PVB19 infection. This case indicates that PVB19 may be one of the causative agents of facial nerve palsy.

## 1. Introduction

Human parvovirus B19 (PVB19) is well-known as a causative pathogen of various pathological conditions, such as erythema infectiosum, arthritis, and hemolytic anemia [1]. Neurological manifestations caused by PVB19 infection, including encephalitis, meningitis, and neuropathy, have been recently reported [2]. The diagnosis of PVB19 infection is made by the detection of anti-PVB19 immunoglobulin M (IgM) antibodies and PVB19 DNA in the serum or cerebral spinal fluid (CSF) [2]. Only one case of peripheral facial palsy with PBV19 infection, which was diagnosed by serology, has been reported in the literature [3]. Here, we report the first case of peripheral facial palsy associated with PVB19 infection in which the diagnosis of PVB19 infection was confirmed by PCR and serology.

## 2. Case Presentation

A 19-month-old boy presented with facial paralysis. His physical growth and development had been good, and he had no history of hospitalization for infections or any diseases. He and his family had no clinical history of immunosuppressive disease. A day before admission, his mother noticed that the left side of his face was not moving normally. Upon visual examination, it was noted that the left eye was not able to close entirely and the left corner of the mouth drooped, especially when he laughed or cried. Furthermore, the left nasolabial fold was absent. He could wrinkle both sides of the forehead. Though a more detailed neurological examination could not be completed because he could not adhere to our instructions, no other neurological symptoms were apparent. Lymphadenopathy was not obvious on palpation. He had neither ear drum swelling nor middle-ear effusion. On visual examination of his entire body, including around both of the ears, no rashes were found. The patient was clinically diagnosed with peripheral facial palsy, and valacyclovir (75 mg/kg for 5 days) and prednisolone (2.1 mg/kg per day for 5 days, then reduced by 0.7 mg/kg every 2 days for a total treatment period of 9 days) were started on admission.

The white blood cell count was 11.4 × 10^3^/*μ*L with 25% neutrophils and 70% lymphocytes. The hemoglobin level was 12.6 g/dL, and the rest of the blood count findings were normal. Peripheral blood smear examination revealed no abnormalities. The laboratory results indicated that the serum chemistry levels were within a normal range. Blood culture was negative. The patient was negative for cytomegalovirus (CMV)-specific IgM antibodies and positive for CMV-specific immunoglobulin G (IgG) antibodies in the serum, indicating that the patient had past CMV infection. The patient was positive for varicella-zoster virus (VZV)-specific IgG antibodies and mumps-specific IgG antibodies in the serum, whereas he was negative for VZV-specific and mumps-specific IgM antibodies, which is attributed to him receiving previous vaccination. The patient was also negative for antibodies against herpes simplex virus (HSV) and Epstein-Barr virus (EBV). Magnetic resonance imaging results of the brain did not show any signs of tumor, infarction, or morphological abnormality of the bilateral inner ear organs and facial nerve.

Facial palsy started to resolve on the 8th day after admission. On the 19th day, erythema appeared on his trunk, and on the 26th day, erythema with a lacy appearance also developed on both of his arms and cheeks, which is consistent with erythema infectiosum. The PVB19-specific serum IgM index measured by enzyme immunoassay was 1.32 (normal < 0.79), and PVB19 DNA (3.9 × 10^4^ copies/mL) was detected in the serum by real-time quantitative PCR [4]. Remission of erythema and peripheral facial palsy were confirmed on the 37th day and 54th day, respectively.

## 3. Discussion

Facial palsy as a clinical presentation is characterized by facial muscular weakness. The causes of acquired peripheral facial palsy in children include idiopathy (Bell's palsy), viral infection, acute otitis media, trauma, and tumor [5]. HSV and VZV are relatively common causative pathogens of peripheral facial palsy, and EBV and CMV are also reported as causative pathogens [5]. On the other hand, PVB19 is not considered as a causative agent in peripheral facial palsy. This is the first case report of facial palsy associated with PVB19 infection confirmed by PCR.

**Figure 1:**
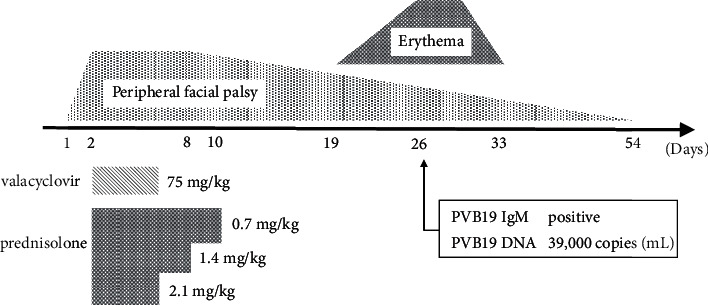
Treatment with valacyclovir and prednisolone started on the second day of peripheral facial palsy. The erythema appeared approximately 3 weeks after peripheral facial palsy was first indicated. PVB19-specific serum IgM was positive and PVB19 DNA was detected in the serum by real-time quantitative PCR on the 26th day. Remission of erythema and peripheral facial palsy were confirmed on the 37th day and 54th day, respectively.

Generally, specific IgM starts to increase 1 week after infection with PVB19, and erythema appears 1-2 weeks later [6]. In some patients, PVB19-specific IgM can persist for 6 months or longer. Therefore, the detection of IgM antibodies may not be conclusive evidence of recent infection. In a previous study, serum PVB19 DNA loads during the acute phase of erythema infectiosum ranged from 4.48 × 10^3^ to 8.31 × 10^6^ copies/mL [6]. The quantity of PVB19 DNA detected in the serum of our patient was the same level to that of the acute phase of erythema infectiosum. It is speculated that the peripheral facial palsy in our patient developed in the early period of PBV19 infection (Figure 1).

The pathophysiology of PVB19-related neurological manifestations remains unclear. Federico reported a case of PVB19-related peripheral facial palsy caused by intraparotid lymphadenitis [3]. However, the patient in our case showed no swelling of the lymph nodes, suggesting that the peripheral facial palsy developed through other mechanisms. Barah cites other pathogenic mechanisms of PVB19 infection, such as direct infection and an indirect mechanism with immune complex or cytokine involvement [7]. There are many types of neurological symptoms of PVB19, and the combination of those mechanisms may cause clinical manifestations.

Unfortunately, thorough neural examination and electroneurography have not been completed for the patient in our case study. Therefore, the objective assessment symptoms may not be enough to elucidate the underlying pathogenic mechanism. We could not perform the detection of virus or antibody in cerebrospinal fluid and antibody titers in paired sera, and the possibility that the PVB19 infection and peripheral facial palsy coincidentally overlapped still exists. However, the results from our case study suggest that PVB19 may be a potential causative pathogen of facial palsy.

## Data Availability

The data used to support the findings of this study are available from the corresponding author upon request.

## References

[1] Kroes A., Jonathan C., William G. P., Steven M. (2016). Parvoviruses. *Infectious Diseases*.

[2] Barah F., Whiteside S., Batista S., Morris J. (2014). Neurological aspects of human parvovirus B19 infection: a systematic review. *Reviews in Medical Virology*.

[3] Martinón-Torres F., Seara M. J. F., Del Río Pastoriza I., Mata M. B., Castro-Gago M. (1999). Parvovirus B19 infection complicated by peripheral facial palsy and parotitis with intraparotid lymphadenitis. *The Pediatric Infectious Disease Journal*.

[4] Shibata Y., Kitajima N., Kawada J. (2005). Association of Cytomegalovirus with infantile hepatitis. *Microbiology and Immunology*.

[5] Ciorba A., Corazzi V., Conz V., Bianchini C., Aimoni C. (2015). Facial nerve paralysis in children. *World Journal of Clinical Cases*.

[6] Ishikawa A., Yoto Y., Tsugawa T., Tsutsumi H. (2014). Quantitation of human parvovirus B19 DNA in erythema infectiosum and aplastic crisis. *Journal of Medical Virology*.

[7] Barah F., Vallely P. J., Cleator G. M., Kerr J. R. (2003). Neurological manifestations of human parvovirus B19 infection. *Reviews in Medical Virology*.

